# Supplementary figures and images for: Effects of Human Leukocyte Antigen DRB1 Genetic Polymorphism on Anti-Cyclic Citrullinated Peptide (ANTI-CCP) and Rheumatoid Factor (RF) Expression in Rheumatoid Arthritis (RA) Patients
Source: Int J Mol Sci. 2023 Jul 27;24(15):12036. doi: 10.3390/ijms241512036 (PMC10418683; doi:10.3390/ijms241512036)

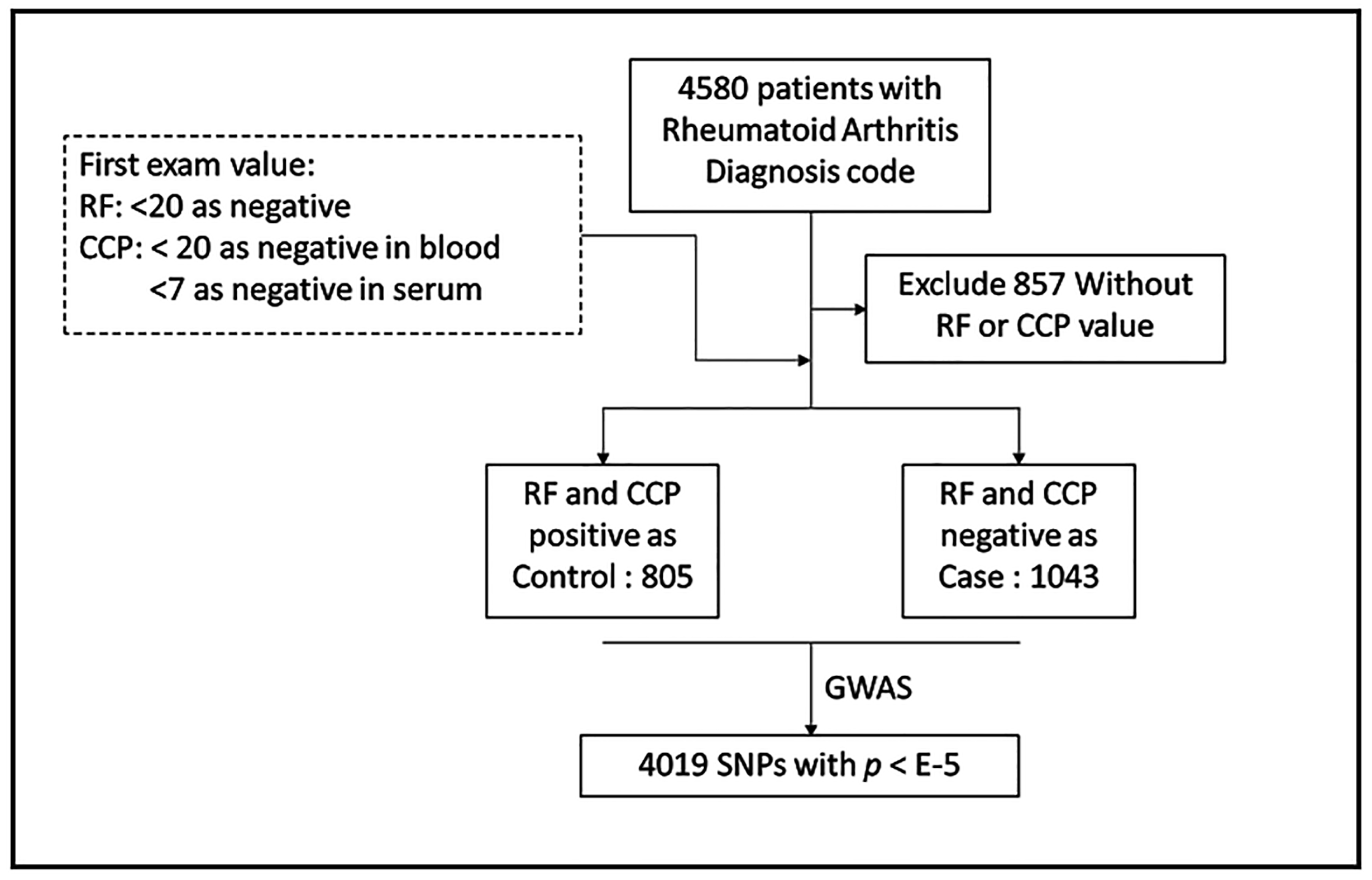

Supplement: Supplementary file 1 [file ijms-24-12036-s001.zip › S7_Figure S1_20230316.tif]
